# Risk factors of immune checkpoint inhibitor-associated acute kidney injury: evidence from clinical studies and FDA pharmacovigilance database

**DOI:** 10.1186/s12882-023-03171-9

**Published:** 2023-04-22

**Authors:** Pengwei Chen, Jianhong Zhu, Yanchun Xu, Qiuyan Huang, Jianan Su, Ziqing Gao, Min Feng

**Affiliations:** 1 Department of Nephrology, Sun Yat-sen Memorial Hospital, Sun Yat-sen University, Guangzhou, 510120 China; 2 Guangdong Provincial Key Laboratory of Malignant Tumor Epigenetics and Gene Regulation, Sun Yat-sen Memorial Hospital, Sun Yat-sen University, Guangzhou, 510120 China; 3 Department of Pharmacy, Sun Yat-sen Memorial Hospital, Sun Yat-sen University, Guangzhou, 510120 China

**Keywords:** Immune checkpoint inhibitor, Acute kidney injury, Systematic review, FDA Adverse Event Reporting System, Real-world pharmacovigilance

## Abstract

**Background:**

Several risk factors of immune checkpoint inhibitors (ICIs)-associated acute kidney injury (AKI) have been reported sporadically. To identify the risk factors of ICIs-associated AKI in a large-scale population, therefore we conducted a systematic review and a real-world retrospective study.

**Methods:**

We search literature concerning risk factors of ICIs-associated AKI in ClinicalTrials.gov and electronic databases (PubMed, Cochrane Library, Embase) up to January 2022. Meta-analysis was performed by using odds ratios (ORs) with 95%CIs. In a separate retrospective pharmacovigilance study by extracting data from US FDA Adverse Event Reporting System (FAERS) database, disproportionality was analyzed using the reporting odds ratio (ROR).

**Results:**

A total of 9 studies (5927 patients) were included in the meta-analysis. The following factors were associated with increased risk of ICIs-associated AKI, including proton pump inhibitors(PPIs) (OR = 2.07, 95%CI 1.78–2.42), angiotensin-converting enzyme inhibitors (ACEIs)/ angiotensin receptor blockers (ARBs) (OR = 1.56, 95%CI 1.24–1.95), nonsteroidal anti-inflammatory drugs (NSAIDs) (OR = 1.29, 95%CI 1.01–1.65), diuretics (OR = 2.00, 95%CI 1.38–2.89), diabetes mellitus (OR = 1.28, 95%CI 1.04–1.57), genitourinary cancer (OR = 1.46, 95%CI 1.15–1.85), combination therapy of ICIs (OR = 1.93, 95%CI 1.25–2.97) and extrarenal immune-related adverse events(irAEs) (OR = 2.51, 95%CI 1.96–3.20). Furthermore, analysis from FAERS database verified that concurrent exposures of PPIs (ROR = 2.10, 95%CI 1.91–2.31), ACEIs/ARBs (ROR = 3.25, 95%CI 2.95–3.57), NSAIDs (ROR = 3.06, 95%CI 2.81–3.32) or diuretics (ROR = 2.82, 95%CI 2.50–3.19) were observed significant signals associated with AKI in ICIs-treated patients.

**Conclusions:**

Concurrent exposures of PPIs, ACEIs/ARBs, NSAIDs or diuretics, diabetes mellitus, genitourinary cancer, combination therapy, and extrarenal irAEs seem to increase the risk of AKI in ICIs-treated patients.

**Supplementary Information:**

The online version contains supplementary material available at 10.1186/s12882-023-03171-9.

## Introduction

Strategies with immune checkpoint inhibitors (ICIs) to treat malignancy have been adopted with a significant improvement in patients' prognosis in recent years. However, ICIs might induce a series of immune-related adverse events(irAEs) due to the unrestricted activation of the immune system and the off-target mode of these drugs [1]. Among the irAEs, an increased risk of acute kidney injury(AKI) was reported with ICIs [2].The incidence of ICIs-associated AKI was 0.8%-4.7% obtained from randomized controlled trials(RCTs) and observational studies [2–6]. Though the incidence of ICIs-associated AKI is not high, it usually leads to discontinuation of the suspicious drugs and add-on immunosuppressive therapy [7, 8], which may add complexity to the anti-tumor therapeutic course.

Several risk factors of ICIs-associated AKI have been reported recently, including the concurrent drugs of proton pump inhibitors (PPIs), angiotensin-converting enzyme inhibitors (ACEIs)/ angiotensin receptor blockers (ARBs) or diuretics, combination therapy with different ICIs, use of ipilimumab or pembrolizumab, coexisting chronic kidney disease (CKD) or low estimated glomerular filtration rate(eGFR), hypertension, the combination of other irAEs and so on [3–5, 9–14]. Early recognition of risk factors may help to reduce the incidence of AKI in ICIs-treated patients. But the above results were only based on every single research.

To identify the risk factors of ICIs-associated AKI in a large-scale population, therefore we conducted a systematic review of observational studies and a real-world study by extracting data on concurrent drugs from the US Food and Drug Administration (FDA) Adverse Event Reporting System (FAERS) database.

### Patients and methods

#### Study design and data sources

Firstly, a meta-analysis of observational studies was conducted to investigate the risk factors of AKI in patients treated with ICIs. Secondly, a real-world analysis was performed by extracting data from the FAERS database to further verified the risk of concurrent drugs of ICI-associated AKI in a large-scale population.

### Systematic review and meta-analysis

This study was registered prospectively in PROSPERO (CRD42021293326). The meta-analysis was performed according to the Preferred Reporting Items for Systematic Reviews and Meta-analysis (PRISMA) statement [15].

### Search strategy

The search for literature was conducted in the following databases: PubMed, Embase, the Cochrane Library, and ClinicalTrials.gov. We performed a systematic literature review of observational studies about ICIs-associated AKI (up to 10th January 2022). The terms searched for literature were that combined “immune checkpoint inhibitor” or “ipilimumab” or “tremelimumab” or “pembrolizumab” or “nivolumab” or “cemiplimab” or “atezolizumab” or “durvalumab” or “avelumab” with “acute kidney injury” or “AKI” or “acute renal failure” or “renal failure acute”.

### Inclusion and exclusion criteria

The inclusion criteria were as follows: 1) observational studies that compared clinical data of patients with versus without AKI after ICIs administration. The definitions and severity of AKI in each included study were demonstrated in supplementary table 1; 2) literature published in English language. Articles that did not meet our requirements (systematic reviews, case reports, case series, comments, protocols, animal studies, and in vitro studies) or lack of full text were excluded, as well as those that did not report the baseline characteristics. We also removed duplicate publications and articles comparing ICIs-associated AKI with AKI caused by other reasons.

### Data extraction and qualitative assessment

After duplicate records were removed, all abstracts were screened by two independent reviewers (P.C and J.Z), and potential eligible articles were searched for full texts. These two review authors also independently extracted data from each study that fulfilled all the criteria. Discrepancies were resolved by a third investigator (M.F). The following information was collected: 1) Sample size; 2) Baseline characteristics: median age with interquartile range (IQR) or mean age with standard deviation (SD), gender, race, baseline eGFR; 3) ICI regimens; 4) Cancer types (melanoma, genitourinary cancer, lung cancer, head and neck cancer); 5) Comorbidities such as diabetes, hypertension, congestive heart failure, chronic obstructive pulmonary disease(COPD), liver disease; 6) Concurrent medications of PPIs, ACEIs/ARBs, nonsteroidal anti-inflammatory drugs(NSAIDs) and diuretics. Quality assessment of each included study was simultaneously performed by two authors(P.C and J.Z) with the Newcastle–Ottawa Scale (NOS) [16]. Discrepancies were resolved by a third investigator (M.F).

### Statistical analysis

Risk factors associated with ICIs-associated AKI were assessed with odds ratio (OR) with 95% confidence interval (CI). The I^2^ statistic was used to present between-study heterogeneity, which of > 50% was considered substantial heterogeneity. Random-effect model was utilized when significant heterogeneity existed. Statistical analyses were performed with STATA software (version 16.0).

### Pharmacovigilance study

The real-world pharmacovigilance study was based on the US FAERS database, which defines adverse drug reactions using Preferred Terms from the Medical Dictionary for Regulatory Activities (MedDRA, version 23.1). We only used the MedDRA Preferred Term “Acute kidney injury” to identify AKI cases as previous studies [17–19]. Moreover, brand names or generic names of ICIs, along with names of targeted concurrent drugs (PPIs, ACEIs/ARBs, NSAIDs, and diuretics) were exploited to identify interested records. Brand names and generic names of all searched drugs were shown in supplementary table 2. Data included in our study is recorded from January 2011 to September 2021. Information was extracted including reporter, age, gender, reported year, reported country, ICI drug name, concurrent medication, the indication of ICIs, and AKI event.

Disproportionality analysis was applied to assess whether AKI was differentially reported in ICIs-treated patients with or without target concurrent drugs. Reporting odds ratio (ROR) was utilized as reported previously [18, 20]. ROR was marked as a significant signal when the lower limit of the 95% CI (ROR_025_) exceeded 1 and with at least 3 reports of AKI simultaneously. Statistical analyses were performed with Microsoft Excel (2021, Microsoft).

## Results

### Systematic review and meta-analysis

#### Study selection

**Fig. 1:**
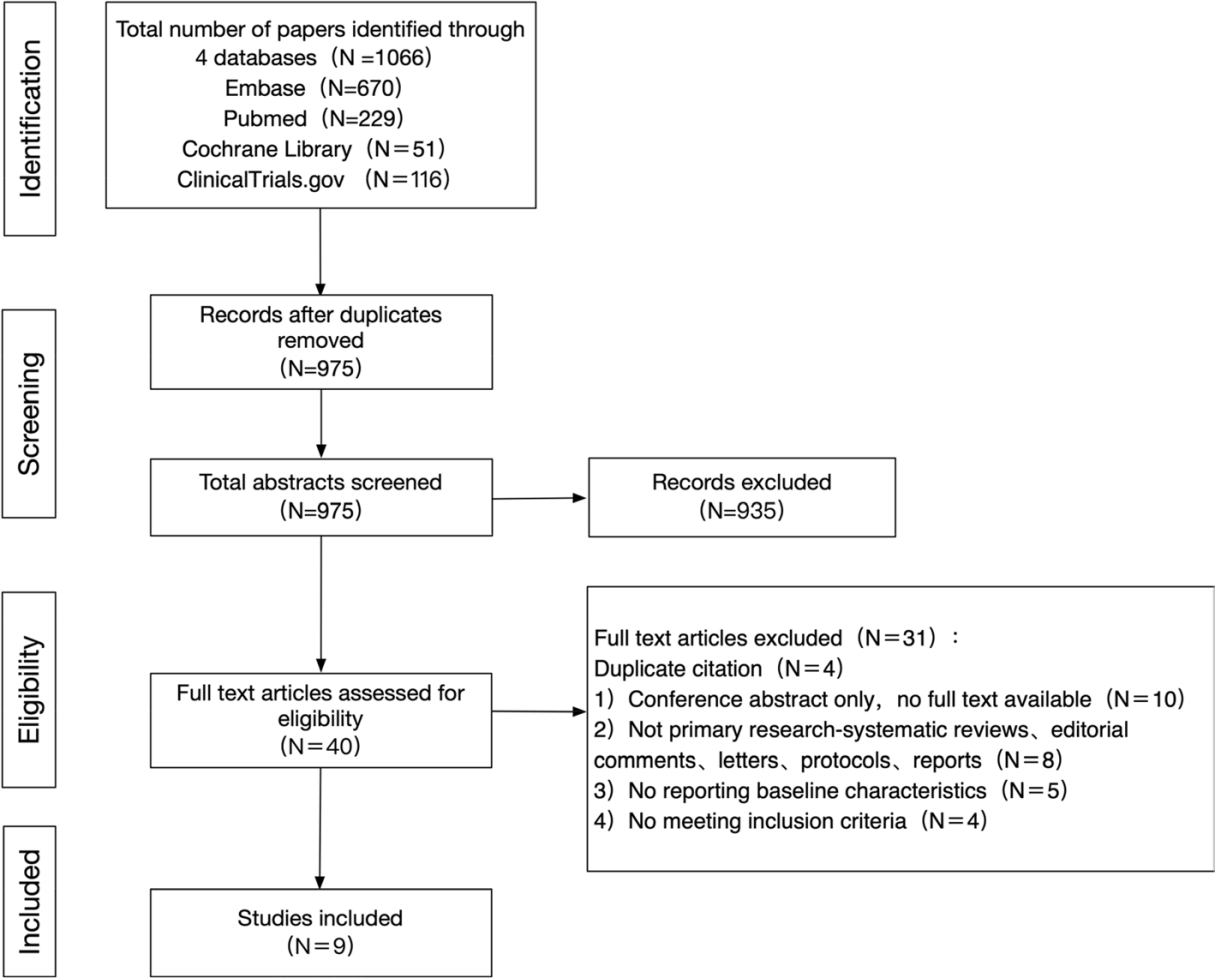
Flow chart of study selection for systemic review

As demonstrated in Fig. 1, the initial search yielded 1066 articles. After duplicate records were removed, the number of studies was reduced to 975, then 935 studies were excluded based on titles and abstracts. Full text of 40 articles was reviewed for further investigation. Finally, 9 studies reporting a total of 5927 ICIs-treated cancer patients were included in our systematic review and meta-analysis.

### Study characteristics

Detailed characteristics of the included studies were summarized in Table 1. Among the 9 eligible studies, 3 studies [3, 5, 12] were reported from the United States, 1 from Canada [10], 1 from France [11], 1 from Japan [13], 1 from Netherlands[4], 1 from North America (including the United States and Canada) [9], and the other from multiregional centers (including North America, Europe, and Asia) [14].

**Table 1:** Characteristics of included studies

Study	Year	Author's country	Sample Size, *N*	Male, *N*(%)	Age, Years, Mean ± SD or Median(IQR)	Baseline eGFR, mL/min/1.73 m^2^, Mean ± SD or Median(IQR)	ICIs-related AKI, *N* (%)	Cases underwent renal biopsy, N (%)
Meraz-Muñoz [10]	2020	Canada	309	186(60.2)	61(51–69)	88(75–99)	51(16.5)	6(11.8)
Abdelrahim [3]	2021	USA	1664	1098(66.0)	NA	NA	72(4.3)	25(34.7)
Seethapathy [5]	2019	USA	1016	616(60.6)	63 ± 13	82 ± 22	169(16.6)	1(0.6)
Cortazar [9]	2020	USA	414	254(61.4)	NA	NA	138(33.3)	60(43.5)
Koks [4]	2021	Netherlands	676	420(62.1)	64(53–71)	90(75–101)	96(14.2)	1 (1.0)
Shimamura [13]	2021	Japan	152	114(75.0)	67 ± 10	72(55–87)	27(17.8)	1 (3.7)
Stein [11]	2020	France	239	132(55.2)	68(58.5–77)	84(70–94)	41(17.1)	3(7.3)
Seethapathy [12]	2020	USA	599	298(49.7)	65 ± 13	88 ± 26	36(6.0)	1(2.8)
Gupta [14]	2021	USA	858	517(60.3)	NA	NA	429(50.0)	151(35.2)

*ICI* Immune checkpoint inhibitor, *AKI* Acute kidney injury, *SD* Standard deviation, *IQR* Interquartile range, *NA* Not available

### Risk of bias assessment

Of these 9 included studies, 5 studies attained a score of 8 with NOS method, 2 studies of 7, 1 study of 6, and 1 study of 5 (Table 2).

**Table 2:** Quality assessment of eligible studies with the Newcastle–Ottawa Scale

Study	Selection				Comparability	Outcome			Scores
	1	2	3	4	5	6	7	8	
Meraz-Muñoz A 2020 [10]	✔	✔	✔		✔✔	✔	✔	✔	8
Abdelrahim 2021 [3]			✔	✔	✔✔	✔	✔	✔	7
Seethapathy 2019 [5]	✔	✔	✔		✔✔	✔	✔	✔	8
Cortazar 2020 [9]	✔		✔		✔✔	✔	✔	✔	7
Koks 2021 [4]	✔	✔	✔		✔✔	✔	✔	✔	8
Shimamura 2021[13]	✔	✔	✔		✔✔	✔	✔	✔	8
Stein 2020 [11]			✔		✔✔	✔	✔	✔	6
Seethapathy 2020 [12]			✔		✔	✔	✔	✔	5
Gupta 2021 [14]	✔	✔	✔		✔✔	✔	✔	✔	8

1 Representativeness of the exposed cohort

2 Selection of the non-exposed cohort

3 Ascertainment of exposure

4 Demonstration that outcome of interest was not present at start of study

5 Comparability of cohorts on the basis of the design or analysis

6 Assessment of outcome

7 Was follow-up long enough for outcomes to occur

8 Adequacy of follow up of cohorts

### Risk factors of ICI-associated AKI

**Fig. 2:**
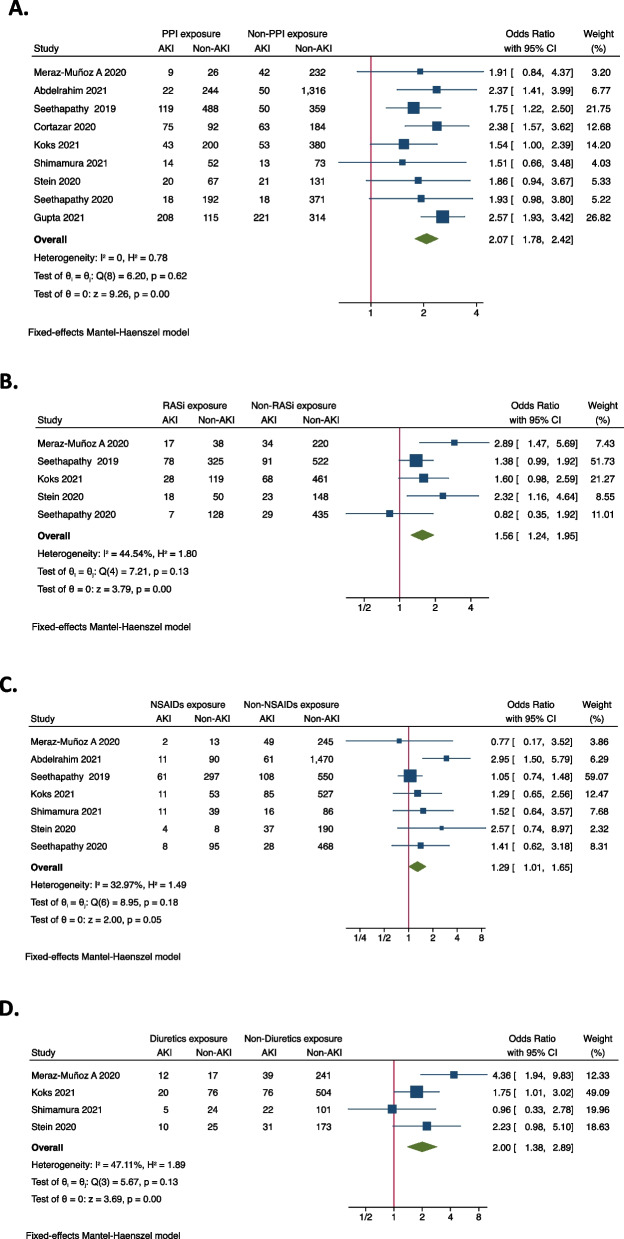
Forest plot of associations between concurrent drugs exposures with risk AKI in patients with ICIs. *PPI* Proton pump inhibitors, *ACEI* Angiotensin-converting enzyme inhibitor, *ARB* Angiotensin receptor blocker, *NSAID* Nonsteroidal anti-inflammatory drug, *AKI* Acute kidney injury, *ICIs* Immune checkpoint inhibitors

**Fig. 3:**
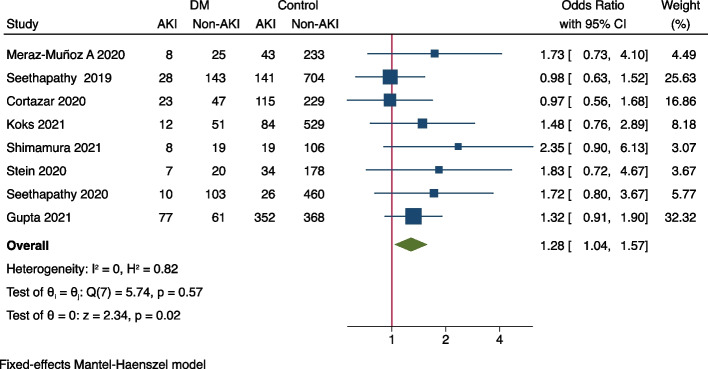
Forest plot of association between DM and AKI in ICIs-treated patients. *DM* Diabetes mellitus, *AKI* Acute kidney injury, *ICIs* Immune checkpoint inhibitors

**Fig. 4:**
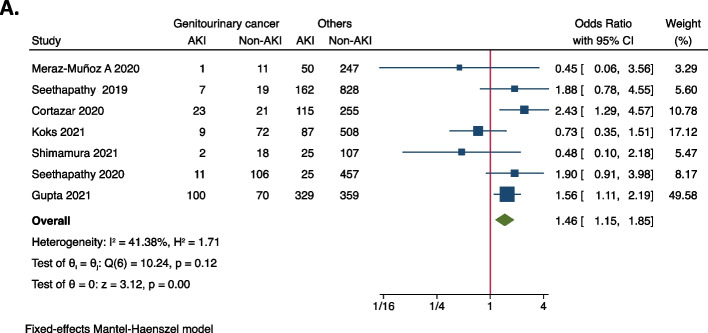
Forest plot of associations between genitourinary cancer and AKI in ICIs-treated patients. *AKI* Acute kidney injury, *ICIs* Immune checkpoint inhibitors

**Fig. 5:**
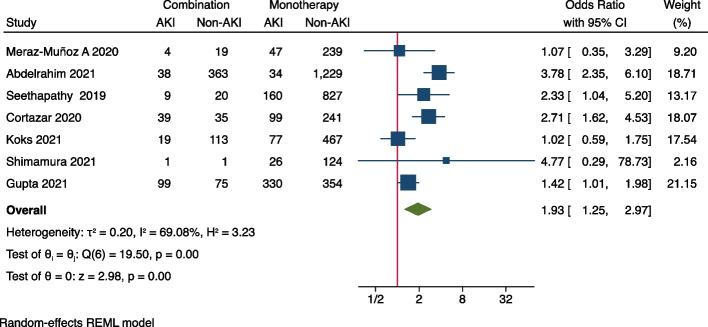
Forest plot of associations between combination therapy and AKI in ICIs-treated patients. *AKI* Acute kidney injury, *ICIs* Immune checkpoint inhibitors

**Fig. 6:**
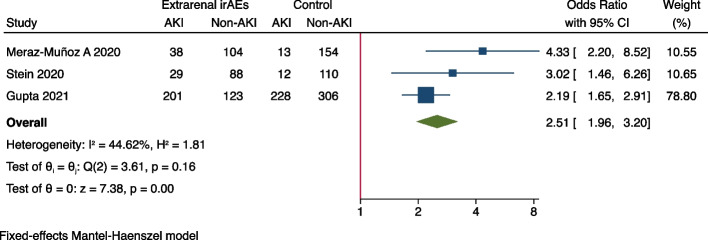
Forest plot of associations between extra-renal irAEs and AKI in ICIs-treated patients. *irAEs* Immune checkpoint inhibitor related adverse events, *AKI* Acute kidney injury, *ICIs* Immune checkpoint inhibitors

Meta-analysis was performed to identify risk factors of ICI-associated AKI. As demonstrated in Fig. 2, concurrent exposures of PPIs (OR = 2.07, 95%CI 1.78–2.42), ACEIs/ARBs (OR = 1.56, 95%CI 1.24–1.95), NSAIDs (OR = 1.29, 95%CI 1.01–1.65), and diuretics (OR = 2.00, 95%CI 1.38–2.89) were all associated with the increased occurrence of AKI in ICIs-treated patients. As for comorbidities, it was shown that coexisting diabetes mellitus (OR = 1.28, 95%CI 1.04–1.57) seems to increase the risk of ICIs-associated AKI (Fig. 3), while hypertension (OR = 1.30, 95%CI 0.91–1.85), congestive heart failure (OR = 1.26, 95%CI 0.69–2.29), liver disease (OR = 1.13, 95%CI 0.67–1.90) and COPD (OR = 0.63, 95%CI 0.26–1.51) were not significantly associated with AKI (Supplementary Fig. 1). In terms of original cancer, it was found that genitourinary cancer (OR = 1.46, 95%CI 1.15–1.85) was associated with an increased risk of AKI in ICI-treated patients (Fig. 4), whilst lung cancer (OR = 0.80, 95%CI 0.66–0.96) and melanoma didn’t increase the risk (Supplementary Fig. 2). None of any ICI category (anti-PD-1, anti-PD-L1, or anti-CTLA-4) was more likely to cause AKI than the others (Supplementary Fig. 3), but the combination of anti-PD-1/ PD-L1 with anti-CTLA-4 (OR = 1.93, 95%CI 1.25–2.97) was found to be significantly correlated with AKI (Fig. 5). It was also worth noting that extrarenal irAEs (OR = 2.51, 95%CI 1.96–3.20) were correlated with an elevated risk of AKI (Fig. 6). Otherwise, the male sex (OR = 1.00, 95%CI 0.86–1.16) and white race (OR = 0.90, 95%CI 0.70–1.16) showed no correlation with AKI in ICIs-treated patients (Supplementary Fig. 4).

### Pharmacovigilance study of FAERS

#### Descriptive analysis

A total of 135,531 adverse events related to ICIs were extracted from FAERS database up to September 2021, with AKI of 2948. The baseline characteristics of these reports were summarized in Table 3. Cases were majorly reported by healthcare professionals, accounting for 83.6%. Most cases were reported from the United States (41.7%) and Japan (15.5%). Male cases account for 62.1% while old patients (≥ 65 years old) for 53.6%. The most common indication for ICIs was lung cancer (28.6%), followed by melanoma (22.1%) and renal carcinoma (8.0%). With the increasing application of ICIs in clinical practice, the number of reported cases also grows.

**Table 3:** Baseline characteristics of ICIs-treated patients with or without AKI events reported from FAERS database

Characteristics	All cases (*N* = 135,531)	AKI (*N* = 2948)	Non-AKI (*N* = 132,583)
Reporter			
Total data	135,186	2945	132,241
Healthcare professional	112,981(83.6%)	2841(96.5%)	110,140(83.3%)
Non-healthcare professional	22,205(16.4%)	104(3.5%)	22,101(16.7%)
Sex			
Total data	119,777	2810	116,967
Male	74,398(62.1%)	1882(67.0%)	72,516(62.0%)
Female	45,379(37.9%)	928(33.0%)	44,451(38.0%)
Age			
Total data	95,464	2679	92,785
< 65YR	44,321(46.4%)	1074(40.1%)	43,247(46.6%)
≥ 65YR	51,143(53.6%)	1605(59.9%)	49,538(53.4%)
Reported countries			
Total data	135,118	2938	132,180
United States, US	56,363(41.7%)	1112(37.8%)	55,251(41.8%)
Japan, JP	20,905(15.5%)	269(9.2%)	20,636(15.6%)
France, FR	11,520(8.5%)	468(15.9%)	11,052(8.4%)
Germany, DE	7422(5.5%)	278(9.5%)	7144(5.4%)
Canada, CA	4755(3.5%)	58(2.0%)	4697(3.6%)
United Kingdom, GB	3600(2.7%)	157(5.3%)	3443(2.6%)
Australia, AU	3122(2.3%)	55(1.9%)	3067(2.3%)
Italy, IT	3012(2.2%)	63(2.1%)	2949(2.2%)
China, CN	2613(1.9%)	11(0.4%)	2602(2.0%)
Spain, ES	2504(1.9%)	76(2.6%)	2428(1.8%)
Other countries	19,302(14.3%)	391(13.3%)	18,911(14.3%)
Indications			
Total data	135,531	2948	132,583
Lung cancer	38,720(28.6%)	755(25.6%)	37,965(28.6%)
Melanoma	29,989(22.1%)	787(26.7%)	29,202(22.0%)
Renal cancer	10,888(8.0%)	330(11.2%)	10,558(8.0%)
Hepatocellular carcinoma	2768(2.0%)	69(2.3%)	2699(2.0%)
Head and neck cancer	2651(2.0%)	44(1.5%)	2607(2.0%)
Gastric cancer	2436(1.8%)	41(1.4%)	2395(1.8%)
Bladder cancer	2065(1.5%)	97(3.3%)	1968(1.5%)
Colorectal cancer	2034(1.5%)	54(1.8%)	1980(1.5%)
Other cancers	43,980(32.5%)	771(26.2%)	43,209(32.6%)

*ICI* Immune checkpoint inhibitor, *AKI* Acute kidney injury

### Disproportionality analysis

ICIs therapy with concurrent exposures of PPIs (ROR = 2.10, 95%CI 1.91–2.31), ACEIs/ARBs (ROR = 3.25, 95%CI 2.95–3.57), NSAIDs (ROR = 3.06, 95%CI 2.81–3.32) and diuretics (ROR = 2.82, 95%CI 2.50–3.19) were observed significant signals associated with AKI (Table 4).

**Table 4:** Disproportional analysis of concurrent medications with AKI

Category	Total (*N*)	a (*N*)	b (*N*)	c (*N*)	d (*N*)	ROR	ROR_025_	ROR_975_
PPIs exposure								
ICIs as a class	12,676	516	12,160	2432	120,423	2.10	1.91	2.31
Anti-PD-1	6016	323	5693	1492	79,096	3.01	2.66	3.40
Nivolumab	3327	209	3118	976	51,415	3.53	3.03	4.12
Pembrolizumab	2605	113	2492	504	27,364	2.46	2.00	3.03
Cemiplimab	84	1	83	12	317			
Anti-PD-L1	3599	101	3498	362	17,493	1.40	1.12	1.74
Atezolizumab	2400	75	2325	273	10,721	1.27	0.98	1.64
Avelumab	223	4	219	43	1579	0.67	0.24	1.89
Durvalumab	976	22	954	46	5193	2.60	1.56	4.35
Anti-CTLA-4	3061	92	2969	578	23,834	1.28	1.02	1.60
Ipilimumab	2572	80	2492	564	22,914	1.30	1.03	1.65
Tremelimumab	489	12	477	14	920	1.65	0.76	3.60
ACEI/ARBs exposure								
ICIs as a class	9261	548	8713	2400	123,870	3.25	2.95	3.57
Anti-PD-1	4690	332	4358	1483	80,431	4.13	3.65	4.67
Nivolumab	2555	229	2326	956	52,207	5.38	4.63	6.25
Pembrolizumab	2063	100	1963	517	27,893	2.75	2.21	3.42
Cemiplimab	72	3	69	10	331	1.44	0.39	5.37
Anti-PD-L1	2298	85	2213	378	18,778	1.91	1.50	2.42
Atezolizumab	1478	66	1412	282	11,634	1.93	1.47	2.54
Avelumab	209	8	201	39	1597	1.63	0.75	3.54
Durvalumab	611	11	600	57	5547	1.78	0.93	3.42
Anti-CTLA-4	2273	131	2142	539	24,661	2.80	2.30	3.40
Ipilimumab	1967	122	1845	522	23,561	2.98	2.44	3.66
Tremelimumab	306	9	297	17	1100	1.96	0.87	4.44
NSAIDs exposure								
ICIs as a class	14,407	765	13,642	2183	118,941	3.06	2.81	3.32
Anti-PD-1	6539	487	6052	1328	78,737	4.77	4.29	5.31
Nivolumab	3668	377	3291	808	51,242	7.26	6.40	8.25
Pembrolizumab	2721	104	2617	513	27,239	2.11	1.70	2.62
Cemiplimab	150	6	144	7	256	1.52	0.50	4.62
Anti-PD-L1	4120	122	3998	341	16,993	1.52	1.23	1.88
Atezolizumab	2614	94	2520	254	10,526	1.55	1.22	1.97
Avelumab	396	9	387	38	1411	0.86	0.41	1.80
Durvalumab	1110	19	1091	49	5056	1.80	1.05	3.06
Anti-CTLA-4	3748	156	3592	514	23,211	1.96	1.63	2.35
Ipilimumab	3150	146	3004	498	22,402	2.19	1.81	2.64
Tremelimumab	598	10	588	16	809	0.86	0.39	1.91
Diuretics Exposure								
ICIs as a class	5513	305	5208	2643	127,375	2.82	2.50	3.19
Anti-PD-1	2452	163	2289	1652	82,500	3.56	3.01	4.20
Nivolumab	1237	112	1125	1073	53,408	4.96	4.04	6.07
Pembrolizumab	1154	49	1105	568	28,751	2.24	1.67	3.02
Cemiplimab	61	2	59	11	341			
Anti-PD-L1	1702	64	1638	399	19,353	1.90	1.45	2.48
Atezolizumab	1208	44	1164	304	11,882	1.48	1.07	2.04
Avelumab	120	6	114	41	1684	2.16	0.90	5.20
Durvalumab	374	14	360	54	5787	4.17	2.29	7.57
Anti-CTLA-4	1359	78	1281	592	25,522	2.63	2.06	3.35
Ipilimumab	1173	72	1101	572	24,305	2.78	2.16	3.58
Tremelimumab	186	6	180	20	1217	2.03	0.80	5.12

a cases reported of AKI with target concurrent drugs;

b cases reported of non-AKI with target concurrent drugs;

c cases reported of AKI with any other concurrent drugs;

d cases reported of non-AKI with any other concurrent drugs

*PPI* Proton pump inhibitor, *ACEI* Angiotensin-converting enzyme inhibitor, *ARB* Angiotensin receptor blocker, *NSAIDs* Non-steroid anti-inflammatory drug; AKI: acute kidney injury

*ROR* Reporting odds ratio

*ROR025* The lower limit of the 95% confidence interval (CI) of the ROR

*ROR975* The upper limit of the 95% confidence interval (CI) of the ROR

## Discussion

Until now, the present study seems to be the first study to identify risk factors of ICIs-associated AKI by a systematic review of clinical studies and analysis of data from a worldwide pharmacovigilance database. Our study suggested that concurrent drugs exposure (PPIs, ACEIs/ARBs, NSAIDs, and diuretics), coexisting diabetes mellitus, genitourinary cancers, combination therapy of ICIs, and extrarenal irAEs were associated with increased risk of AKI events in ICIs-treated cancer patients.

PPIs have been reported to increase the risk of AKI in the general population with an abundance of evidence [21]. Deposition of PPI metabolite in the renal tubular area and interstitium may cause acute interstitial nephritis and acute kidney injury [22]. In cancer patients with ICIs, the concomitant medication of PPIs was revealed to be a risk factor of ICIs-associated AKI in several publications [3, 5, 9, 14], but negative results were reported as well [4, 10–13]. In our study, both results of the systematic review (including 9 studies) and analysis data from FARES suggested that concomitant medication of PPIs increased the risk of ICIs-associated AKI, indicating the caution of PPI use in cancer patients treated with ICIs. Analyzed from FAERS database, PPIs exposure in drugs of anti-PD1 as a class, anti-PDL1, and anti-CTLA4 had positive signals with AKI. Regarding of individual drugs, PPIs exposure in only nivolumab, pembrolizumab, durvalumab and ipilimumab had significant positive signals with AKI, whilst negative in other ICIs. Owing to certain relatively new drugs (cemiplimab, avelumab, tremelimumab) being approved not for a long time, the clinical results of these drugs were limited, so further investigation is still needed.

ACEIs and ARBs both have effects on vasodilation of the renal efferent arterioles thus causing a reduction of glomerular filtration pressure. During the hypovolemia state, the reduced efferent vascular tone as described above may induce AKI [23]. Nevertheless, the evidence of ACEIs/ARBs directly leading to AKI is lacking [24]. Several guidelines still recommend to withhold ACEIs/ARBs during certain acute states, such as sepsis, hypovolemia, or hypotension [24]. Whether the concomitant use of ACEIs/ARBs is a risk factor of ICIs-associated AKI is still controversial based on the published studies [4, 5, 10–12]. In our study, both of the results suggested that exposure to ACEIs/ARBs is related to the increased risk of ICIs-associated AKI. From the FAERS database, regardless of the ICIs types (anti-PD1, anti-PDL1 or anti-CTLA4), ACEIs/ARBs exposure had significant positive signals with AKI. According to individual drugs, results were nearly similar with PPIs, while nivolumab, pembrolizumab, atezolizumab, and ipilimumab show positive results. These results indicate that ACEIs/ARBs would better be replaced by other anti-hypertensive agents to reduce the risk of AKI when accompanied by ICIs.

NSAIDs were reported to be correlated with an increased risk of AKI in the general population in both children and adults. The possible reasons were as follows: NSAIDs can reduce renal blood flow; and may cause tubular obstruction owing to crystal deposition then induce direct cytotoxicity and cell-mediated immune attack [25, 26]. Among the published studies in which NSAIDs were mentioned, only one study found that patients with NSAIDs exposure had a higher incidence of ICIs-associated AKI [3]. In our study we pooled data from 7 studies and found that NSAIDs exposure was associated with an increased risk of ICIs-associated AKI. Positive signals of AKI were also found in ICIs-treated patients with NSAIDs exposure, based on the analysis from FAERS. Our results indicated that, in order to minimize the risk of AKI, avoidance of NSAIDs and ICIs concurrent use is recommended.

Diuretics are reported to be a risk factor of AKI after liver transplantation and related with a higher risk of perioperative AKI [27, 28]. Diuretics use hasn’t been found to correlate with ICIs-AKI depending on existing publications [4, 10, 11, 13]. Here in our present study, we pooled data from 4 observational studies and found that diuretics use increased the risk of ICIs-AKI, though the number of eligible studies was limited. Moreover, results from FARES also supported our results of the meta-analysis mentioned above. It is implicated when diuretics and ICIs are used together, the occurrence of AKI should be vigilant.

Coexisting diabetes mellitus DM is a risk factor of AKI in a certain status, such as infection with coronavirus disease-19, liver transplantation, percutaneous coronary intervention, and so on [29–31]. However, diabetes hasn’t been verified to be an independent risk factor of AKI in patients who received ICIs treatment from individual observational study until now. Through pooling data from 8 studies, we found that coexisting diabetes increased the risk of AKI in patients who received ICIs treatment. It is indicated that monitoring urinalysis and renal function is necessary for diabetic patients treated with ICIs and with significant importance.

AKI was reported to occur in genitourinary cancers with a relatively high incidence [32–34], partially owing to the obstruction of urinary tract and destruction of the kidney. Genitourinary cancer was regarded as a potential risk factor of AKI in patients treated with ICIs in 7 studies, but no positive results were found. By pooling data from these 7 studies, AKI was prone to occur in ICIs-treated patients with genitourinary cancer. It is implied that special awareness should be paid to this subgroup of patients when treated with ICIs.

A combination of anti-CTLA-4 and anti-PD1/PDL1 was found to be an independent risk factor of ICIs-associated AKI in several studies [3, 9], whereas negative results were found in other researches [4, 5, 10, 13, 14]. By pooling data from 7 studies in our meta-analysis, the combination of ICIs was revealed to correlate with increased risk for AKI. The explanation of this phenomenon may be due to the dual immune checkpoint blockage resulting in enhanced stimulation of autoreactive T cells.

Multisystem irAEs could be induced during the treatment with ICIs, mainly attributed to the unrestricted activation of the immune system and the off-target mode of ICIs [1]. The irAEs could occur in the central nervous system, skin, liver, heart, lungs, musculoskeletal system, gastrointestinal tract, and kidneys [8]. Similar to the published observational studies [10], our research also suggested that extrarenal irAEs were correlated with the increased incidence of AKI. Extrarenal irAEs may reflect the degree of the immune system activated by ICIs, hence increasing the possibility of off-target immune reactions in the kidney.

There still be some limitations in our study. Firstly, all of the eligible studies were retrospective; thus, other possible confounding factors may influence the results. Secondly, a specific risk factor only reported in only one study was excluded in the present study due to it is impossible to pool the results in a meta-analysis. Thirdly, the relation between the factors with positive results and ICIs-related AKI could only be association. Further studies still need to be introduced to clarify the causal relationship. What’s more, reporting to FAERS was voluntary, hence the relationship between concurrent drugs (PPIs, ACEI/ARBs, NSAIDs, and diuretics) exposure in patients with ICIs and suspected adverse event (AKI event) was not clear and definite. Also, differences in the definition of AKI were unclear. Besides, detailed clinical information such as PD-1 status, patient’s baseline renal function, or other risk factors related to AKI were missing. Finally, comparisons of incidence for adverse events through a disproportionality analysis might be influenced by many confounding factors, such as reporting bias and lack of denominator data [35].

Following the increasing use of ICIs, AKI related to these new anti-tumor agents have been reported and prompted more investigations. Based on meta-analysis of observational studies and real-world pharmacovigilance study of FAERS, our results suggested that drugs exposure (PPIs, ACEIs/ARBs, NSAIDs, and diuretics), coexisting diabetes mellitus, genitourinary cancers, combination therapy of ICIs and extrarenal irAEs may increase the risk of AKI events in ICI-treated patients. Future studies are needed to investigate the mechanism and optimal management of ICIs-associated AKI.

## Supplementary Information

**Additional file 1.** Supplementary figure.

**Additional file 2.** Supplementary tables.

## Data Availability

Data from the meta-analysis are freely and publicly available on published literature. Data of real-world pharmacovigilance study are collected from the FDA Adverse Event Reporting System, and can be obtained through the FAERS Public Dashboard website.
