# Supplementary material for: Risk factors of immune checkpoint inhibitor-associated acute kidney injury: evidence from clinical studies and FDA pharmacovigilance database
Source: BMC Nephrol. 2023 Apr 22;24:107. doi: 10.1186/s12882-023-03171-9 (PMC10122540; doi:10.1186/s12882-023-03171-9)
Supplement: Supplementary file 1 — Additional file 1. Supplementary figure. [file 12882_2023_3171_MOESM1_ESM.pptx]

## Slide 1
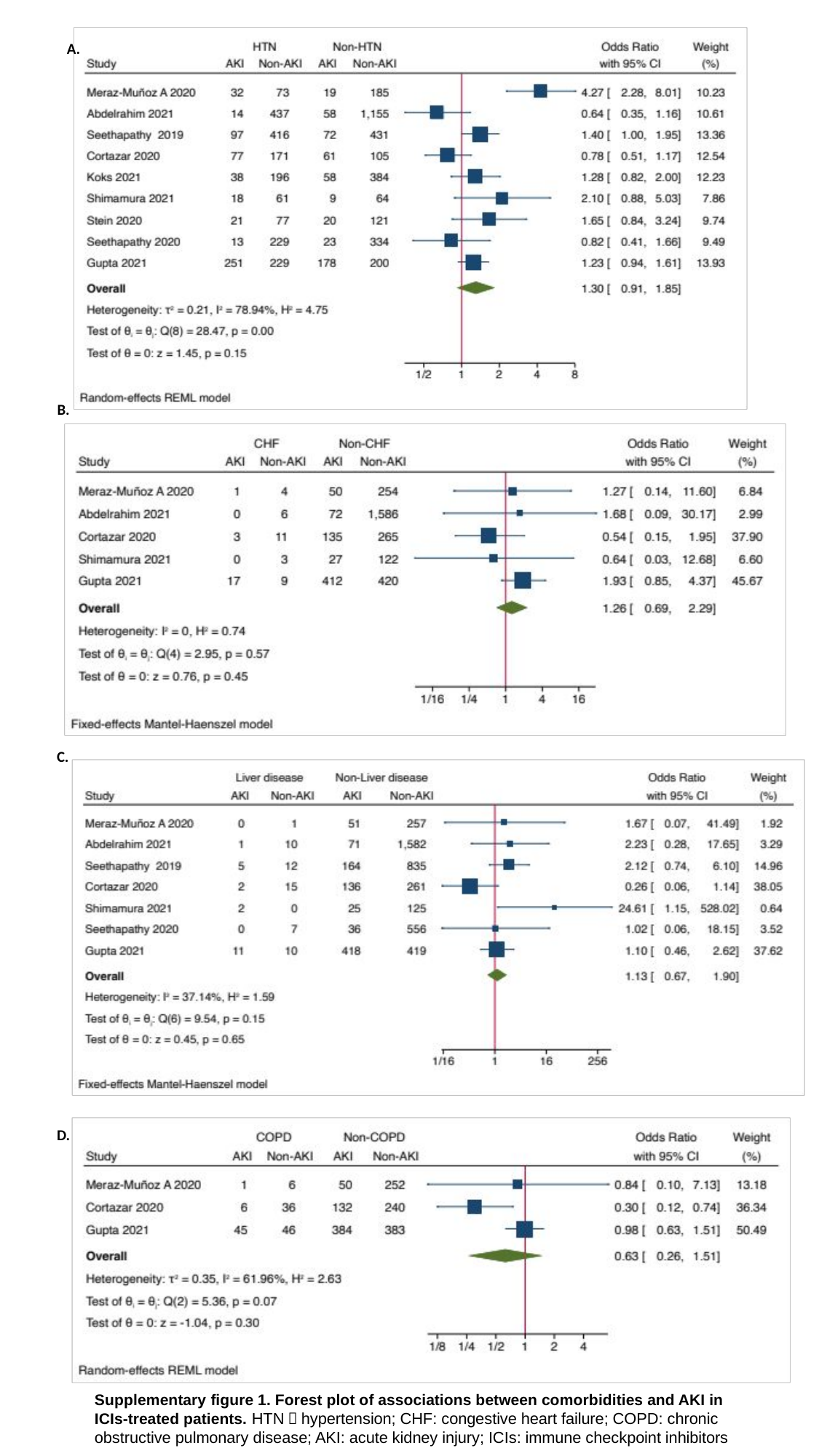

A.
B.
C.
D.
Supplementary figure 1. Forest plot of associations between comorbidities and AKI in ICIs-treated patients. HTN：hypertension; CHF: congestive heart failure; COPD: chronic obstructive pulmonary disease; AKI: acute kidney injury; ICIs: immune checkpoint inhibitors

## Slide 2
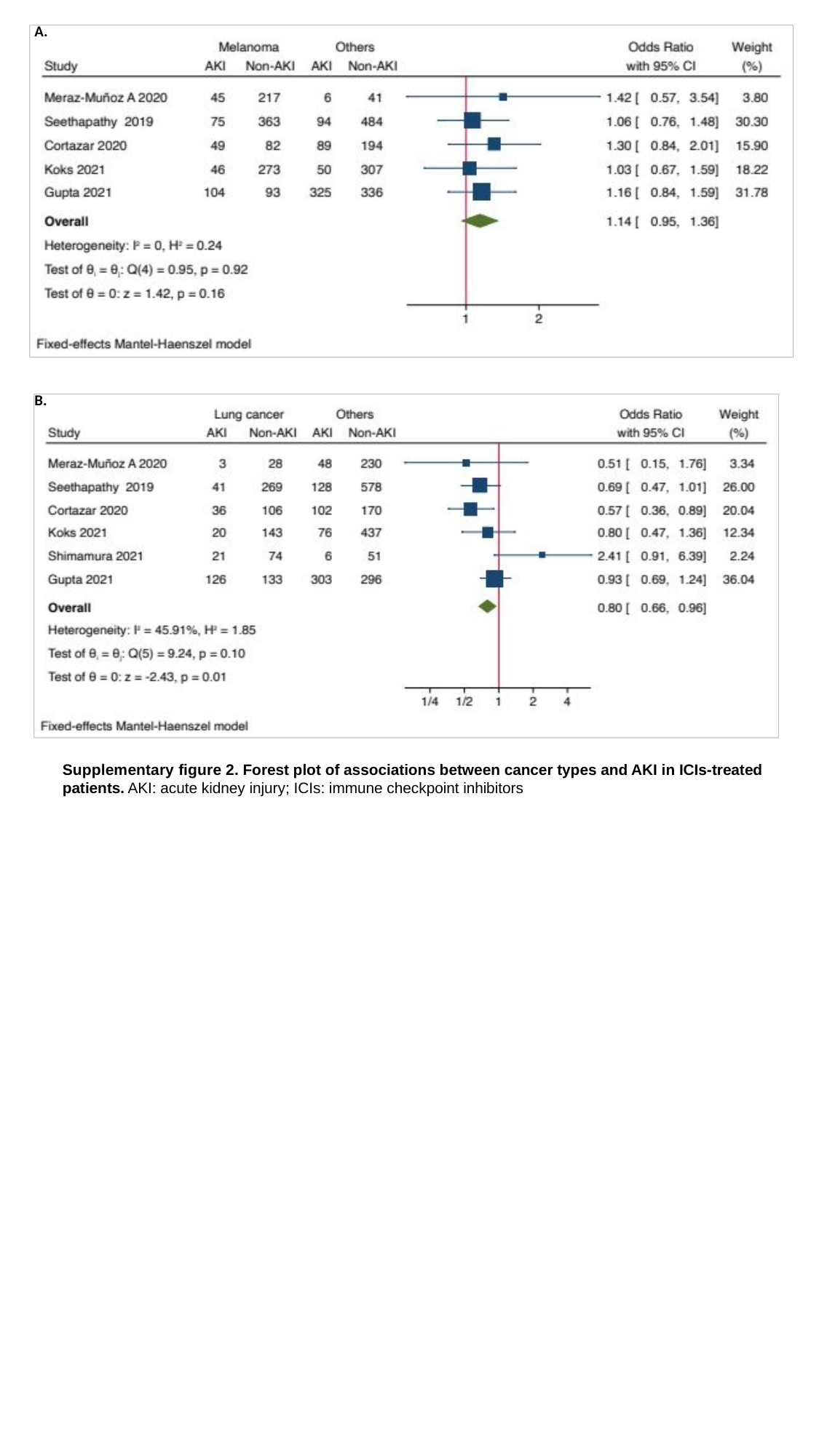

A.
B.
Supplementary figure 2. Forest plot of associations between cancer types and AKI in ICIs-treated patients. AKI: acute kidney injury; ICIs: immune checkpoint inhibitors

## Slide 3
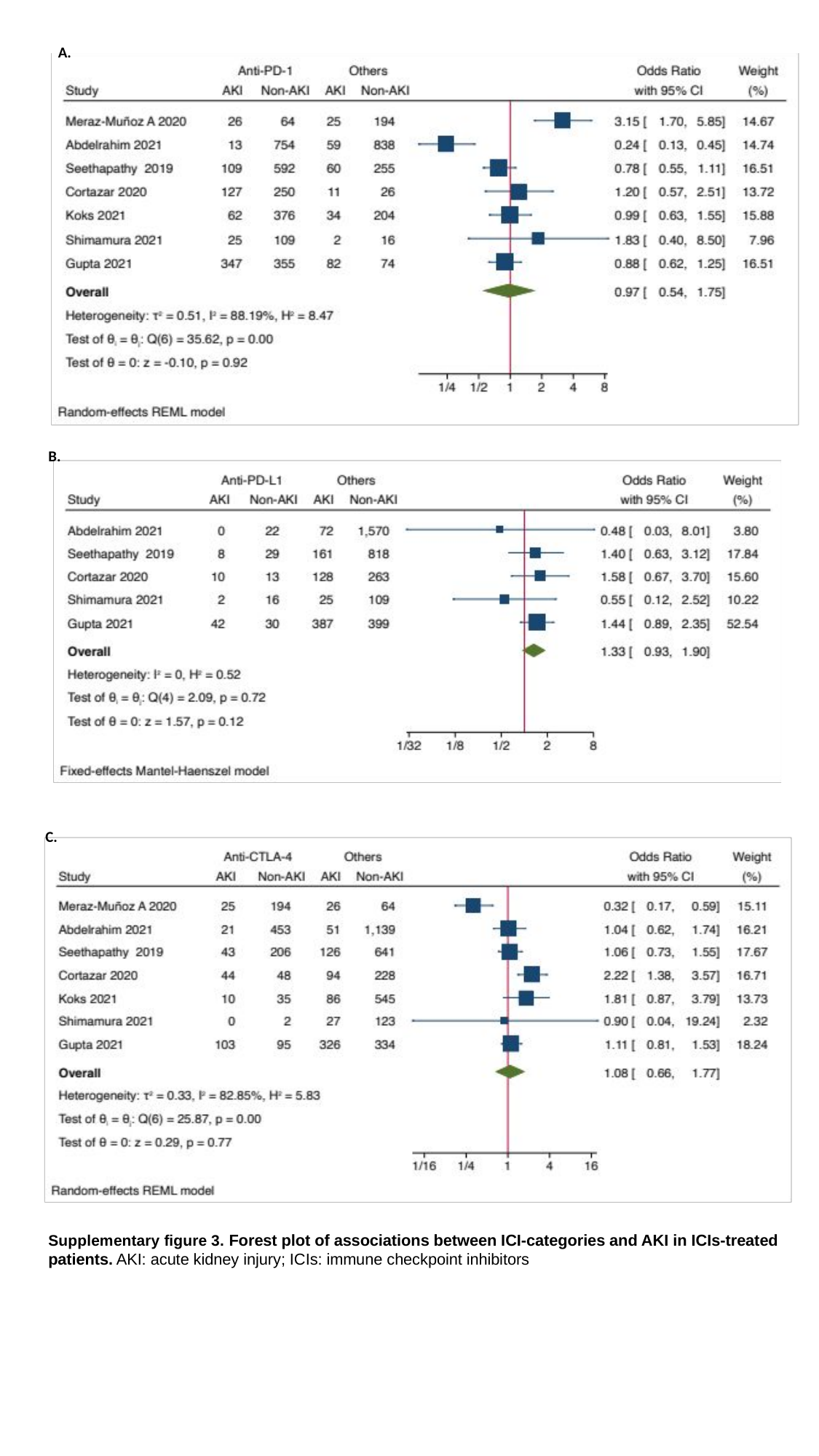

A.
B.
C.
Supplementary figure 3. Forest plot of associations between ICI-categories and AKI in ICIs-treated patients. AKI: acute kidney injury; ICIs: immune checkpoint inhibitors

## Slide 4
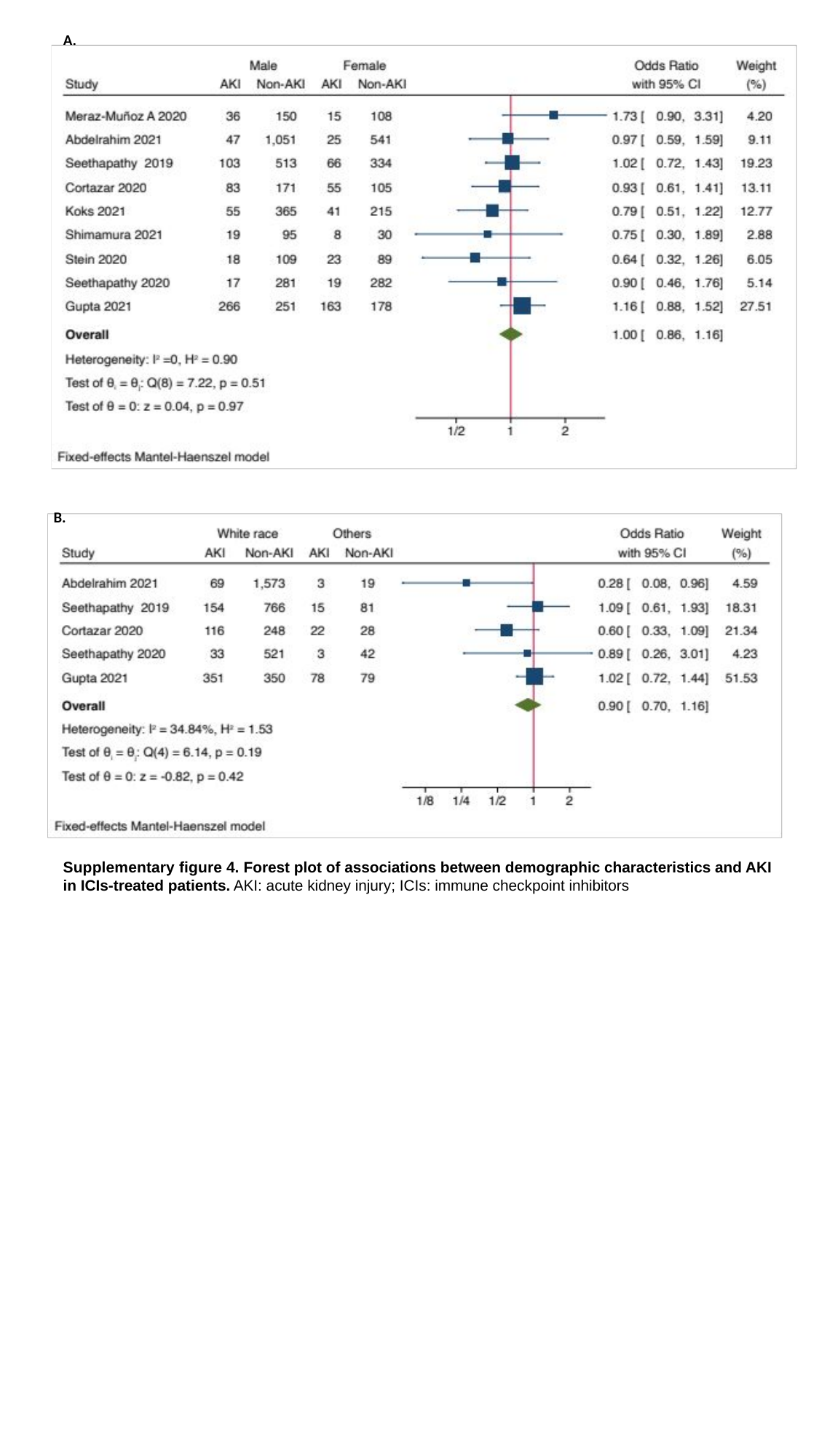

A.
B.
Supplementary figure 4. Forest plot of associations between demographic characteristics and AKI in ICIs-treated patients. AKI: acute kidney injury; ICIs: immune checkpoint inhibitors
