# Supplementary material for: Risk factors of immune checkpoint inhibitor-associated acute kidney injury: evidence from clinical studies and FDA pharmacovigilance database
Source: BMC Nephrol. 2023 Apr 22;24:107. doi: 10.1186/s12882-023-03171-9 (PMC10122540; doi:10.1186/s12882-023-03171-9)
Supplement: Supplementary file 2 — Additional file 2. Supplementary tables. [file 12882_2023_3171_MOESM2_ESM.docx]

**Supplementary table 1 The definitions of AKI in included studies**

| **Study** | **AKI definition** |
| --- | --- |
| Meraz-Muñoz 2020 | AKI was defined and staged according to the KDIGO criteria. |
| Abdelrahim 2021 | AKI was defined as a relative increase in serum creatinine of ≥50% within 7 days. |
| Seethapathy 2019 | AKI was defined as a ≥1.5-fold increase in creatinine from baseline within 12 months of ICI initiation.  AKI severity was staged according to KDIGO criteria. |
| Cortazar 2020 | AKI was defined as at least a doubling of serum creatinine or the requirement for RRT.  AKI severity was staged according to KDIGO criteria. |
| Koks 2021 | AKI was defined and staged according to the KDIGO criteria. |
| Shimamura 2021 | AKI was defined and staged according to the KDIGO criteria. |
| Stein 2020 | AKI was defined and staged according to the KDIGO criteria. |
| Seethapathy 2020 | AKI was defined as a ≥1.5-fold increase in creatinine from baseline.  AKI severity was staged according to KDIGO criteria. |
| Gupta 2021 | (1)an increase in serum creatinine ≥100% from baseline or treatment with RRT; (2) an increase in SCr ≥50% from baseline and at least one of the following: ATIN on kidney biopsy; ICI therapy held for at least one cycle due to concern for ICI-AKI; or treatment with corticosteroids due to concern for ICI-AKI.  AKI severity was staged according to KDIGO criteria. |

AKI : acute kidney injury; KDIGO: Kidney Disease: Improving Global Outcomes; ICI: immune checkpoint inhibitor: RRT: renal replacement therapy; ATIN: acute tubulointerstitial nephritis.

**Supplementary table 2 Brand names and generic names of all searched drugs**

| Category | Generic name | Brand name |
| --- | --- | --- |
| ICI | Nivolumab,Pembrolizumab,Cemiplimab,Atezolizumab,Avelumab,Durvalumab,Ipilimumab,Tremelimumab | / |
| PPIs | Omeprazole,Esomeprazole,Lansoprazole,Pantoprazole,Rabeprazole,Vonoprazan | NEXIUM,PRILOSEC,TALICIA,VIMOVO,YOSPRALA,ZEGERID,DEXILANT,PREVACID, PROTONIX, ACIPHEX |
| ACEI/ARB: | Quinapril, Perindopril,Ramipril, Benazepril,Captopril,Enalapril,Fosinopril,Lisinopril,Trandolapril,Perindopril,Cilazapril,MidApril,Delapril,Valsartan,Candesartan,Irbesartan,Losartan,Eprosartan | ACCUPRIL,ACCURETIC,QUINARETIC,ACEON,ALTACE,LOTENSIN,LOTREL,CAPOTEN,CAPOZIDE,EPANED,LEXXEL,TECZEM,VASERETIC,VASOTEC,MONOPRIL,PRINIVIL,PRINZIDE,QBRELIS,ZESTORETIC,ZESTRIL,MAVIK,TARKA,MOEXIPRIL,UNIRETIC,UNIVASC,PRESTALIA,SPIRAPRIL,RENORMAX,OLMESARTAN,AZOR,BENICAR,TRIBENZOR,BYVALSON,DIOVAN,ENTRESTO,EXFORGE,VALTURNA,ATACAND,AVALIDE,AVAPRO,AZILSARTAN,EDARBI,EDARBYCLOR,HYZAAR,COZAAR,TEVETEN,TELMISARTAN,MICARDIS,TWYNSTA,PREXXARTAN |
| NSAID | Aspirin,Acetaminophen,Indomethacin,Naproxen,Naproxone,Diclofenac,Ibuprofen,Nimesulide,Rofecoxib,Etoricoxib | AGGRENOX,AXOTAL,AZDONE,CODOXY,DARVON,DURLAZA,EQUAGESIC,EXCEDRIN,FIORINAL,INVAGESIC,LANORINAL,MEASURIN,MICRAININ,NORGESIC,NORGESIC-FORTE,ORPHENGESIC,PERCODAN,PRAVIGARDPAC,QGESIC,ROBAXISAL,ROXIPRIN,SOMACOMPOUND,SYNALGOSDC,TALWIN,VAZALORE,VICOPRIN,YOSPRALA,ACEPHEN,ALLAY,ALLZITAL,ANEXSIA,ANOQUAN,APADAZ,BANCAP,BUCET,BUTAPAP,COGESIC,CODRX,DARVOCET,DRIXORAL,DURADYNE,ESGIC,FEMCET,HYPHEN,INJECTAPAP,LORTAB,NEOPAP,NORCET,NORCO,OFIRMEV,OXYCET,PHRENILIN,PROPACET,PROVAL,ROXICET,SEDAPAP,TALACEN,TENCON,TREZX,TRIAD,TRIAPRIN,TYCOLET,TYLENOL,TYLOX,ULTRACET,VICODIN,WYGESIC,XARTEMIS,ZYDONE,ZYFREL,TIVORBEX,INDOLEMMON,INDOCIN,ALEVE,ANAPROX,NAPRELAN,NAPROSYN,TREXIMET,VIMOVO,ARTHROTEC,CAMBIA,CATAFLAM,DYLOJECT,FLECTOR,LICART,PENNSAID,SOLARAZE,VOLTAREN,ZIPSOR,ZORVOLEX,ACHESNPAIN,ADVIL,CALDOLOR,CAPPROFEN,ELIXSURE,MOTRIN,COMBUNOX,DUEXIS,IBUPRIN,IBUPROHM,MEDIPREN,NEOPROFEN,NUPRIN,REPREXAIN,RUFEN,VIOXX,Celecoxib,CELEBREX,CONSENSI,ELYXYB,SEGLENTIS,ARCOXIA |
| Diuretic | Furosemide,Ethacrynic acid,Chlorothiazone,Indapamide,Metolazone,Spironolactone,Eplerenone,Triamterene,Amiloride,Tolvaptan,Acetazolamide,Mannitol ,Sorbitol | LASIX,TORSEMIDE,DEMADEX,SOAANZ,BUMETANIDE,BUMEX,EDECRIN,CHLOROTHIAZIDE,HYDRODIURIL,LOZOL,DIULO,MYKROX,ZAROXOLYN,ALDACTAZIDE,ALDACTONE,CAROSPIR, INSPRA, DYAZIDE,DYRENIUM,MAXZIDE,MIDAMOR,HYDRO-RIDE,MODURETIC,JYNARQUE,SAMSCA, ,DIAMOX,ARIDOL,BRONCHITOL,OSMITROL,RESECTISOL,MICROZIDE |

ICI: Immune checkpoint inhibitor; PPI: proton pump inhibitor; ACEI: angiotensin-converting enzyme inhibitor; ARB: angiotensin receptor blocker; NSAIDs: non-steroid anti-inflammatory drugs
